# Investigations Relating to the Etiology and Prophylaxis of Yellow Fever

**Published:** 1888-06

**Authors:** 


### Editorial.

### INVESTIGATIONS RELATING TO THE ETIOLOGY AND PROPHYLAXIS OF YELLOW FEVER.

AN ADDRESS DELIVERED BEFORE THE COLLEGE OF PHYSICIANS OF PHILADELPHIA, APRIL 21, 1888, BY GEORGE M. STERNBERG, M. D., MAJOR AND SURGEON U. S. ARMY.

A publication, with the above title, in the *Medical News*, of April 28th, presents the points of a report, made in compliance with an appointment by President Cleveland, for an investigation of the methods of inoculation practiced in Brazil and Mexico, by which it is claimed that protection against yellow fever is afforded.

The recognized qualifications of Dr. Sternberg as a bacteriologist and microscopist fitted him pre-eminently for this scientific work, and his familiarity with sanitary matters afforded a reasonable ground to expect that all would be accomplished which could be done by a single individual in developing the facts.

An unbiased judgment, with a due regard to the great interests involved, must be awarded the commissioner in entering upon this very important exploration, and it is evident that much labor and painstaking has been displayed in his examination of the data presented to him in Brazil and Mexico.

The report made to the government, as to the result of his inquiries, is therefore to be regarded as a final disposition of the official proceedings instituted, after repeated appeals had been presented to Congress by the American Public Health Association and the American Medical Association in favor of the appointment of a commission to investigate this subject. Whether the expectations of those interested in a proper solution of this vexed question are satisfactorily met by the investigation of this matter by a single individual, from his own stand-point, remains yet to be seen in the light of further developments. But for the present we must accept the dictum of Dr. George M. Sternberg, that "there is no satisfactory evidence that the method of inoculation practiced by Dr. Domingos Friere has any prophylactic value," and that "the claims of Dr. Carmona y Valle, of Mexico, to have discovered the specific cause of yellow fever have likewise no scientific basis, and he has failed to demonstrate the protective value of his proposed method of prophylaxis."

The patient experimentation of these would-be philanthropists, tending to the conclusion that a great discovery had been made for the benefit of mankind, is thrown to the winds by two sentences of a scientist representing the United States Government.

It may not be out of place to interpolate a few matters in regard to which medical men and many citizens of this country feel some curiosity. Why Dr. Sternberg should have gone to Brazil to prosecute his investigation as to yellow fever inoculation in the months of June and July, when the coast cities are known to be free from that disease, calls for some explanation which does not appear in this address. This is more surprising, as at that time yellow fever prevailed on the coast of Mexico, where it was not found when his visit was made subsequently. Hence, "unfortunately, the opportunity did not offer itself to make cultures from the various organs, obtained at the earliest possible moment after death."

Another point of interest to those acquainted with the fact is that Dr. Sternberg's visit to Rio de Janeiro should have been made when Dr. Domingos Friere was absent in Paris, and that he should have been in conference with Dr. Araujo Goes, the avowed antagonist of Friere, for a month previous to the return of the latter. His inability to see clearly what was pointed out to him afterwards by Friere may have resulted from some influence upon his vision from looking through the glasses of Dr. Goes.

The most puzzling development connected with this investigation was the device resorted to by Dr. J. B. Hamilton, Surgeon-General of the Marine Hospital Service, to prevent the appointment of Drs. Guiteras and Lane as additional commissioners for the examination of the claims of yellow fever inoculation in Brazil and Mexico. After the final action of the American Medical Association at Chicago, recommending the appointment of two additional commissioners, passed over a motion to lay on the table and a motion to reconsider, an unparliamentary proceeding was instituted by Dr. Hamilton to effect the rescinding of the resolutions on the morning of the last day when comparatively few of the members of the Association were present.

These circumstances, taken into consideration with many others occurring in the course of the proceedings before Congress, calculated to thwart the object of securing a commission of three competent persons for this undertaking, may well cause doubts as to the reliance to be placed upon the report of Dr. Sternberg to the government. The desideratum is to learn the facts in regard to prevention of yellow fever by inoculation, and the decision of this does not rest upon a scientific recognition of the cryptococcus xanthogenicus as the source of yellow fever, but upon a verification of the protective inoculation with the cultivated virus.

